# Pulmonary function and health-related quality of life 1-year follow up after cardiac surgery

**DOI:** 10.1186/s13019-016-0491-2

**Published:** 2016-07-08

**Authors:** Elisabeth Westerdahl, Marcus Jonsson, Margareta Emtner

**Affiliations:** Faculty of Medicine and Health, School of Health Sciences, Örebro University, Örebro, Sweden; Faculty of Medicine and Health, School of Medical Sciences, Örebro university, Örebro, Sweden; Department of Medical Sciences, Respiratory Medicine and Allergology, Uppsala University, Uppsala, Sweden

**Keywords:** Cardiac surgery, Health-related quality of life, Postoperative complications, Respiratory function tests, Thoracic surgery

## Abstract

**Background:**

Pulmonary function is severely reduced in the early period after cardiac surgery, and impairments have been described up to 4–6 months after surgery. Evaluation of pulmonary function in a longer perspective is lacking. In this prospective study pulmonary function and health-related quality of life were investigated 1 year after cardiac surgery.

**Methods:**

Pulmonary function measurements, health-related quality of life (SF-36), dyspnoea, subjective breathing and coughing ability and pain were evaluated before and 1 year after surgery in 150 patients undergoing coronary artery bypass grafting, valve surgery or combined surgery.

**Results:**

One year after surgery the forced vital capacity and forced expiratory volume in 1 s were significantly decreased (by 4–5 %) compared to preoperative values (*p* < 0.05). Saturation of peripheral oxygen was unchanged 1 year postoperatively compared to baseline. A significantly improved health-related quality of life was found 1 year after surgery, with improvements in all eight aspects of SF-36 (*p* < 0.001). Sternotomy-related pain was low 1 year postoperatively at rest (median 0 [min-max; 0–7]), while taking a deep breath (0 [0–4]) and while coughing (0 [0–8]). A more pronounced decrease in pulmonary function was associated with dyspnoea limitations and impaired subjective breathing and coughing ability.

**Conclusions:**

One year after cardiac surgery static and dynamic lung function measurements were slightly decreased, while health-related quality of life was improved in comparison to preoperative values. Measured levels of pain were low and saturation of peripheral oxygen was same as preoperatively.

## Background

Postoperative pulmonary complications are still a major cause of adverse outcome after cardiac surgery and contribute considerably to morbidity and mortality. Pulmonary function is significantly reduced in the immediate postoperative period and the reason for the impairment is multifactorial and today not fully understood [1].

In addition to effects of anaesthesia and intraoperative events, postoperative pain, physical impairments and activity restrictions in the postoperative period may further aggravate the pulmonary impairment [2]. Many efforts to enhance postoperative pulmonary recovery have been made. Postoperative care, early mobilization, physiotherapy interventions and cardiac rehabilitation programs are aimed at supporting the recovery course following cardiac surgery and reducing the negative impact of surgery. Even though an increased level of physical activity compared to preoperative level has been reported as early as 2 months after surgery, pulmonary function is still not restored at this point [3]. Impairment of pulmonary function has been described up to 4–6 months after cardiac surgery [4, 5]. However, evaluation of pulmonary function in a long-term perspective is lacking.

A review by Noyez et al. [6] concluded that there was a lack of evaluations of quality of life in a long-term perspective after cardiac surgery, and that only nine out of 29 studies had presented preoperative health-related quality of life (HRQoL) data to compare with postoperative data. Three of these studies had a follow-up period up to 1 year, showing progressive improvements in Short-Form Health Survey (SF-36) scores after mitral valve surgery [7], coronary artery bypass grafting [8] and valve replacement [9]. To our knowledge, no studies have evaluated pulmonary function and its possible associations with health-related outcomes in a longer perspective. Therefore, the aim of this study was to describe pulmonary function and HRQoL 1 year after cardiac surgery, and to determine any associations between pulmonary function measurements and subjective breathing and coughing ability.

## Methods

### Patients

**Fig. 1:**
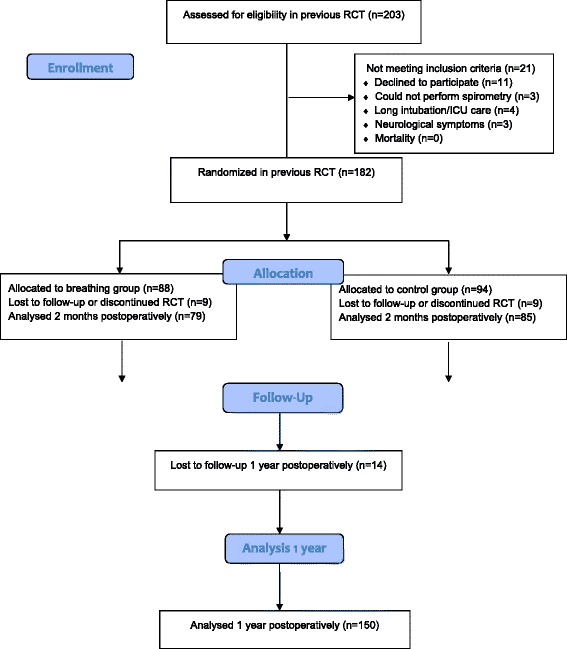
Study flowchart of the patients in the study

A total of 164 patients undergoing cardiac surgery at Örebro University Hospital, Örebro, Sweden, selected from a previous two-centre investigation [10], were considered for the study, i.e. only patients from one centre was invited to be re-evaluated 1 year postoperatively. Both patients subjected to deep breathing exercises and controls were included from the initial randomisation, with no significant difference between groups regarding pulmonary function 2 months postoperatively [10]. Of these, 14 declined to participate in the 1-year follow-up and so the final sample consisted of 150 patients (Fig. 1). The patients underwent coronary artery bypass grafting (CABG), valve surgery (aortic, mitral or tricuspidalis valve lesions) or combinations of CABG and valve surgery. Patients were not included if they had intubation time > 24 h; postoperative severe hemodynamic, pulmonary, neurological complications; requirement for dialysis; sternum infections or instability; mental health disorders; or any other complications which could affect their opportunity to perform pulmonary function tests. The study was approved by the Regional Ethical Review Board in Uppsala, Sweden (2007/160) and informed consent was obtained from each patient. The main trial was registered at ClinicalTrials.gov (NCT01282671; URL: www.clinicaltrials.gov).

### Surgical and postoperative treatment

The surgery was performed through a median sternotomy. For CABG saphenous vein grafts and/or the left or right internal mammary artery graft were used. During anaesthesia and following surgery, all patients inspired oxygen at a concentration of 40–80 %. The pericardium, the mediastinum and occasionally one or both pleura were drained, usually less than 24 h after surgery. Postoperatively, patients were artificially ventilated with a positive end-expiratory pressure of 5–10 cmH_2_O. After extubation, all patients received pain relief according to their needs, following routine postoperative protocol. The patients were recommended to use 1 g paracetamol four times daily for as long as needed after discharge. Postoperative routines, early mobilization, breathing exercises and chest physical therapies have been described elsewhere [10]. The patients performed hourly deep breathing exercises from the first postoperative day until discharge and patients randomized to the intervention group in the main study performed home-based breathing exercises 2 months postoperatively. Demographic and descriptive data were collected from medical records.

### Measurements

All measurements were assessed preoperatively (the day before surgery, or the week before if operated on a Monday) and 1 year after surgery. The pulmonary function measurements were performed on a Jaeger MasterScreen PFT/Bodybox (Intramedic AB, Bålsta, Sweden) by biomedical scientists at the Department for Clinical Physiology at Örebro University Hospital. Measurements were performed according to the recommendations of the American Thoracic Society and European Respiratory Society. Vital capacity (VC), forced vital capacity (FVC), forced expiratory volume in one second (FEV_1_), FEV1/FVC, peak expiratory flow (PEF), inspiratory capacity (IC), functional residual capacity (FRC), residual volume (RV) and total lung capacity (TLC) were assessed. The patients were examined in a sitting position, and a nose-clip was used. Predicted values were related to age, sex, and height [11]. Patients with FEV1/FVC ratio < 0.7 were defined as having airway obstruction/airflow limitation according to GOLD criteria (www.goldcopd.org).

Saturation of peripheral oxygen (SpO_2_) was measured using a pulse oximeter device (Rad-5v; Masimo, Irvine, CA, USA).

HRQoL was assessed using the Swedish version of the SF-36 (first version), a self-administered 36-item questionnaire covering eight different domains: physical functioning, physical role functioning, bodily pain, general health, vitality, social functioning, emotional role functioning, and mental health [12]. Raw points are transformed into a score from 0 to 100 for each dimension, with 100 reflecting the best possible HRQoL.

The patients rated their dyspnoea on the modified Medical Research Council dyspnoea scale (mMRC) from 0 to 4 and their subjective ability to take deep breaths and cough on a numeric rating scale (NRS) from 0 (can perform with no difficulty) to 10 (cannot/impossible to perform). One year after surgery they were asked to report sternotomy-related wound pain at rest, while taking a deep breath and while coughing, using a NRS from 0 (no pain) to 10 (worst imaginable pain).

### Statistical analysis

Data are presented as mean ± SD [95 % confidence interval of the difference] or median [min-max]. Preoperative and postoperative values were compared using Student’s paired *t*-test (pulmonary function) or the Wilcoxon signed-rank test (HRQoL, subjective breathing and coughing ability and dyspnoea). Pulmonary function was reported as absolute values and as percentages of predicted values. P-values refer to the difference between the preoperative and 1 year postoperative percentage predicted values. The Spearman rank correlation test was used to identify associations between pulmonary function (VC, FVC, FEV1 and IC) in percentage of predicted values and dyspnea, subjective breathing and coughing ability. All results refer to two-sided tests, and p-values less than 0.05 were considered significant. Version 15.0 of the SPSS software package (SPSS Inc., Chicago, IL, USA) was used for the statistical analysis.

## Results

A total of 150 patients completed pulmonary function tests and questionnaires before and 1 year after cardiac surgery. Demographic and surgical variables are presented in Tables 1 and 2.

**Table 1:** Preoperative demographic variables

Variables (*n* = 150)	
Male/female (n)	123/27
Age (years)	66 ± 9
BMI (kg/m^2^)	27 ± 4
Never smoked/ex-smoker/smoker (n)	57/85/8
Airway obstruction/airflow limitation (n)	37
Diabetes mellitus (n)	29
Hypertension (n)	77
NYHA class I-II/III-IV/missing value (n)	64/75/11
EuroSCORE	4 ± 2
Left ventricular ejection fraction (%)	57 ± 10

Patients with preoperative FEV1/FVC < 0.70 were defined as having airway obstruction

Data are presented as mean ± SD or number (n) of patients

*BMI* body mass index, *NYHA* New York Heart Association, *FEV1* forced expiratory volume in 1 s, *FVC* forced vital capacity

**Table 2:** Surgical variables

Variables (*n* = 150)	
Isolated CABG/Isolated valve/CABG + valve (n)	54/59/37
On pump/Off pump	147/3
Operation time (h)	3 ± 1
ECC time (min)	112 ± 53
AoO time (min)	84 ± 46
Peripheral anastomoses per patient	3 ± 1
Central anastomoses per patient	1 ± 1
LIMA graft/RIMA graft (n)	62/0
Left pleural space entered (n)	31
Bilateral pleural space entered (n)	28
Postoperative mechanical ventilation (h)	6 ± 4
Duration of anaesthesia (h)	10 ± 3

Duration of anaesthesia = start of anaesthesia to extubation

Data are presented as mean ± SD or number (n) of patients

*CABG* coronary artery bypass grafting, *ECC* extracorporeal circulation, *AoO* aortic occlusion time, *LIMA* left internal mammary artery, *RIMA* right internal mammary artery

Before surgery, the patients had normal pulmonary function in relation to reference values (mean FVC: 103 ± 18 % of predicted and mean FEV1: 98 ± 19 % of predicted). One year after surgery VC, FVC, FEV1, FEV1/VC (%), PEF and FRC were significantly reduced (by 2–5 %) compared to preoperative values, while no significant impairment was found in IC, RV and TLC. Absolute values, percentage predicted and percentage changes from preoperative values are shown in Table 3. SpO_2_ was unchanged one year postoperatively compared to baseline (97 ± 1 % vs. 97 ± 2 %, *p* = 0.396).

**Table 3:** Pulmonary function data before and 1 year after cardiac surgery (*n* = 150)

	Before surgery	1 year after surgery	1 year after surgery	Preoperative to postoperative change in pulmonary function (% predicted)	
	Mean ± SD	Mean ± SD	Mean ± SD	CI for the change [95 % CI] *P*-value	
	(% predicted)	(% predicted)	(% preoperative values)		
VC (l)	4.1 ± 1.0	4.0 ± 1.0	97.5 ± 8.3 %	−1.7 ± 9.0 % [−3.1 – -0.2]	0.025
	(104.4 ± 16.8)	(102.8 ± 17.2)	98.7 ± 9.0 %		
FVC (l)	3.9 ± 1.0	3.7 ± 1.0	96.4 ± 10.8 %	−2.9 ± 11.0 % [−4.6 – -1.1]	0.002
	(103.5 ± 18.1)	(100.6 ± 18.6)	97.8 ± 11.3 %		
FEV_1_ (l)	2.9 ± 0.8	2.7 ± 0.8	94.8 ± 10.6 %	−4.1 ± 10.5 % [−5.8 – -2.4]	<0.001
	(98.2 ± 19.5)	(94.2 ± 20.5)	96.1 ± 11.0 %		
FEV_1_/VC (%)	71.0 ± 8.5	68.9 ± 8.8	97.3 ± 7.5 %	−2.5 ± 6.5 % [−3.6 – -1.5]	<0.001
	(94.0 ± 11.1)	(91.5 ± 11.5)	97.4 ± 7.2 %		
PEF (l/min)	542.4 ± 145.7	526.4 ± 136.1	98.2 ± 13.1 %	−2.8 ± 16.2 % [−5.4 – -0.2]	0.035
	(117.2 ± 26.6)	(114.4 ± 23.7)	99.0 ± 14.6 %		
IC (l)	3.1 ± 0.8	3.1 ± 0.8	101.4 ± 14.5 %	1.7 ± 15.7 % [−0.8 – 4.3]	0.181
	(108.5 ± 20.8)	(110.2 ± 22.9)	102.4 ± 15.1 %		
FRC (l)	3.4 ± 0.8	3.3 ± 0.8	97.6 ± 11.5 %	−3.0 ± 11.2 % [−4.8 – -1.2]	0.001
	(98.6 ± 19.7)	(95.6 ± 19.0)	97.6 ± 11.3		
RV (l)	2.5 ± 0.7	2.5 ± 0.6	101.0 ± 16.9 %	−1.3 ± 15.7 %[−3.9 – 1.2]	0.314
	(102.4 ± 24.4)	(101.1 ± 22.6)	100.2 ± 16.3 %		
TLC (l)	6.5 ± 1.3	6.4 ± 1.3	98.8 ± 7.8 %	−1.5 ± 11.5 % [−3.4 – 0.3]	0.107
	(99.3 ± 13.5)	(97.8 ± 15.7)	98.8 ± 11.7 %		

Data are presented as mean ± SD [95 % CI of the difference]. *P*-values refer to the difference between preoperative and postoperative values (percentage predicted). *P*-value < 0.05 was considered significant

*CI* confidence interval, *FEV*_*1*_ forced expiratory volume in 1 s, *FVC* forced vital capacity, *FRC* functional residual capacity, *IC* inspiratory capacity, *PEF* peak expiratory flow, *RV* residual volume, *TLC* total lung capacity, *VC* vital capacity

A significantly improved HRQoL was found 1 year after surgery, with improvements in all eight domains of SF-36 (Table 4). Sternotomy-related pain (NRS) was low 1 year postoperatively at rest (0 [0–7]), while taking a deep breath (0 [0–4]), and while coughing (0 [0–8]).

**Table 4:** SF-36 scores before and 1 year after cardiac surgery (*n* = 133)

	Before surgery	1 year after surgery	Preoperative to postoperative change in SF-36 scores	
	Mean ± SD	Mean ± SD,	CI for the change [95 % CI] *P*-value	
	Md (min-max)	Md (min-max)		
PF	65.0 ± 24.5	81.7 ± 20.7	16.7 [12.8–20.5]	<0.001
	70 (5–100)	90 (10–100)		
RP	39.8 ± 43.0	79.0 ± 33.7	39.2 [31.1–47.3]	<0.001
	25 (0–100)	100 (0–100)		
BP	69.4 ± 27.6	83.6 ± 22.3	14.1 [9.1–19.2]	<0.001
	74 (12–100)	100 (0–100)		
GH	64.2 ± 18.1	77.4 ± 17.7	13.2 [10.3–16.0]	<0.001
	65 (15–100)	82 (25–100)		
VT	54.8 ± 23.7	73.6 ± 18.6	18.8 [15.0–22.6]	<0.001
	55 (0–100)	80 (25–100)		
SF	78.8 ± 24.3	92.8 ± 13.8	14.0 [9.9–18.1]	<0.001
	88 (13–100)	100 (50–100)		
RE	67.2 ± 42.2	82.3 ± 33.8	15.2 [7.1–23.2]	<0.001
	100 (0–100)	100 (0–100)		
MH	74.5 ± 19.7	85.8 ± 15.2	11.3 [7.9–14.7]	<0.001
	80 (12–100)	92 (36–100)		

Data are presented as mean ± SD and median (min-max) [95 % CI of the difference]. *P*-value < 0.05 was considered significant

*BP* bodily pain, *CI* confidence interval, *GH* general health, *Md* median, *MH* mental health, *PF* physical functioning, *RE* emotional role functioning, *RP* physical role functioning, *SF* social functioning, *VT* vitality. Incomplete/missing data *n* = 17

Median [min-max] values for dyspnoea were 1 [0–4] preoperatively and 1 [0–3] a year after surgery; a significant reduction (z = −6.2, *p* < 0.001). Subjective ability to take deep breaths was improved from baseline to 1 year postoperatively (0 [0–9] vs. 0 [0–8]; z = −3.8, *p* < 0.001) and the ability to cough was also improved (1 [0–8] to 0 [0–8]; z = −3.4, *p* = 0.001). One year after surgery, a weak negative correlation was found between pulmonary function (percentage of predicted) and dyspnea, breathing and coughing ability (Table 5).

**Table 5:** Correlation matrix of the associations between pulmonary function (% predicted) and dyspnoea, ability to take deep breaths and cough 1 year after cardiac surgery (*n* = 150)

Pulmonary function (percentage predicted)	Dyspnoea (mMRC)	Ability to take deep breaths (NRS)	Ability to cough (NRS)
VC	−0.17 (*p* = 0.054)	−0.14 (*p* = 0.105)	−0.15 (*p* = 0.090)
FVC	−0.19* (*p* = 0.038)	−0.16 (*p* = 0.065)	−0.18* (*p* = 0.043)
FEV1	−0.18 (*p* = 0.050)	−0.19* (*p* = 0.030)	−0.19* (*p* = 0.030)
IC	−0.09 (*p* = 0.340)	−0.19* (*p* = 0.029)	−0.15 (*p* = 0.087)

mMRC, Modified Medical Research Council dyspnea scale; NRS: numeric rating scale

*P-value < 0.05 was considered significant

## Discussion

The findings of this study show that pulmonary function is significantly decreased 1 year after cardiac surgery, with a reduction of 4–5 % in FVC and FEV1 compared to preoperative values. No reduction in peripheral oxygen saturation was found, and HRQoL was improved one year after surgery, as expected. One year after surgery, a more pronounced decrease in pulmonary function was associated with impairments regarding dyspnoea and subjective breathing and coughing ability.

Patients undergoing cardiac surgery develop restrictive lung volumes, impaired ventilatory mechanics, decreased lung compliance and increased breathing effort postoperatively [13]. Atelectasis, lower respiratory tract infection and ventilatory insufficiency are considered the leading causes of postoperative morbidity. Although 30-day mortality after cardiac surgery has decreased during recent years, the 1-year mortality has not changed [14]. There are limited publications dealing with long-term changes in respiratory function after cardiac surgery. An early study by Braun et al. [15] revealed significant reductions in volumes (11–17 %), diffusion capacity (7–11 %) and arterial oxygen tension (12 %) in 19 patients 2 weeks after CABG [15]. Van Belle found that significant reductions in TLC, VC and FEV1 (*n* = 18) persisted 6 weeks after CABG [16]. Pulmonary function deterioration has been found up to 3 months [17], 3.5 months [4] and 4 months [5] after cardiac surgery. A description of pulmonary function after cardiac surgery as long as 1 year postoperatively has not previously been presented.

The reasons for the pulmonary impairment found 1 year postoperatively in the present study are unknown. Alterations in chest wall mechanics induced by surgery and sternotomy may persist for a long time. Opening of the pleural space has also been suggested as a reason for postoperative dysfunction. In addition, the cardiopulmonary bypass procedure has a negative influence on pulmonary function. Changes in blood cortisol and c-reactive protein concentrations during the 3 first days after surgery has been described to be independently associated with larger reductions in pulmonary function [1]. Secondary to cardiac dysfunction, a low postoperative cardiac output may lead to ventilation-perfusion mismatch, and pulmonary oedema may further deteriorate pulmonary function. Respiratory impairments after open mitral valve surgery have been described 3 months after surgery, despite haemodynamic and functional improvements [18].

Postoperative phrenic nerve paralysis and associated diaphragmatic dysfunction could also be a reason for impaired pulmonary function after surgery [19]. Postoperative pain may cause hypoventilation and insufficient cough. In the present study, pain could not explain the decreased \pulmonary function. Sternotomy-related pain was low 1 year after surgery, with median value 0, even while taking a deep breath or coughing.

The aim of cardiac surgery is to prolong life, relieve symptoms and improve functional status. Thus, improvement in HRQoL is considered to be an important outcome. Significantly improved HRQoL was found 1 year after surgery in the present study, with improvements in all domains of SF-36. This is in agreement with the results of Gjeilo et al., who found improvement in all subscales of SF-36 1 year after cardiac surgery; and even 5 years after surgery, seven of the eight subscales were still higher than before surgery [20]. Improvement in all eight health domains of SF-36 scores 1 year after cardiac surgery has also been described by Kurfirst et al. [21]. Noyez recently proposed that quality of life after cardiac surgery is overestimated, at least for older, high-risk patients and patients with low preoperative values [22]. This could apply to our study, since the patients reported low quality of life preoperatively. HRQoL is an important aspect in assessing outcomes of any surgical intervention. We used a generic HRQoL questionnaire, since there is no disease-specific validated instrument for the cardiac surgery population. Although no consensus exists about the precise definition of HRQoL, many instruments have been developed to assess it. Patientsʼ quality of life may be affected in many ways, including symptoms of angina and heart failure, limited exercise capacity and psychological stress. In cardiac surgery patients, the SF-36 has been reported to be better than the Nottingham Health Profile questionnaire in terms of internal consistency, ceiling effect and sensitivity to change [23].

The patients and the study from which they were selected have been described previously [10]. No emergency patients or patients who had previous cardiac or lung surgery or kidney failure requiring dialysis were included in the sample, and even if old and high-risk patients were included, this limitation might underestimate postoperative lung function impairments. In patients with severe hemodynamic, pulmonary or neurological complications, longer surgery or sternum-related problems, who were excluded from the study, one could expect even more pronounced decrease in pulmonary function. For natural reasons of ageing, a decrease in pulmonary function is to be expected after 1 year. The normal reduction in pulmonary function according to age is a decrease in VC and FEV1 about 20–30 mL per year in non-smokers [11]; for this reason, we calculated differences between the preoperative and postoperative values as percentages of predicted values.

Our results show that pulmonary function, including VC, FVC, FEV1, FEV%, PEF and FRC, was still significantly reduced 1 year after cardiac surgery. This indicates that impairment of pulmonary function after open heart surgery is long lasting, and may even be permanent. However, the patients’ subjective experience of breathing and coughing ability 1 year after surgery was comparable to preoperative values, and the patients reported less dyspnoea. We found significant associations between an impaired pulmonary function and more pronounced dyspnoea as well as decreased subjective breathing and coughing ability 1 year after cardiac surgery. The correlation was weak (correlation coefficients - 0.09 to −0.19) and the clinical significance if this finding needs to be further investigated.

The decrease in pulmonary function 1 year postoperatively was of somewhat smaller extent than found in a previous study by us, where we reported a decrease in spirometric values of 6–13 % 4 months after CABG [5]. In an earlier study by Shenkman et al., an even more pronounced decrease in pulmonary function was described 3.5 months after cardiac surgery; FVC by an average of 24 % and FEV1 by an average of 23 % [4]. Surgical and anaesthetic techniques have been developed during the years and might be an explanation for less reduction in postoperative pulmonary function found in the present study.

The patients received basic postoperative chest physiotherapy as conventionally used at the Department of Cardiothoracic Surgery including early mobilization and deep breathing exercises during the first postoperative days, and cardiac rehabilitation offered to most patients postoperatively. No method of postoperative pulmonary therapy has been distinguished as superior in preventing or treating long-term changes in pulmonary function after cardiac surgery and clinical practice varies regarding recommendations on therapy after discharge [10, 24].

Decreased length of hospitalization for cardiac surgery patients poses challenges to the possibilities of providing interventions that will facilitate optimal recovery. Physical activity is beneficial in both the prevention and treatment of patients with ischaemic heart disease, and patients undergoing CABG have demonstrated improved functional capacity 2 years after surgery [25]. We have previously presented positive effects in pulmonary function in more physically active cardiac surgery patients [3].

One limitation in this study is that we did not include any acute surgery patients, and we also excluded patients with previous cardiac or lung surgery. Thus, our sample might be healthier than the true population. We have no information on the patients who declined participation in the study, who could potentially be in a worse condition than patients who agreed to take part. Overestimation of postoperative HRQoL is important to consider, since the dropouts may have been those with a low quality of life. We used a numeric rating scale to measure pain; however, assessing pain is difficult because of its complex and subjective nature.

This is the first study to evaluate pulmonary function and HRQoL in a long-term postoperative perspective. We followed the patients for 1 year after cardiac surgery, which is longer than previously described in the literature. The postoperative recovery of impairment in pulmonary function is delayed for at least 1 year after cardiac surgery. Pulmonary dysfunction with impairment of pulmonary function and oxygenation is a serious problem in the early postoperative period after cardiac surgery, but it is questionable whether the reductions of 4–5 % which persist 1 year postoperatively are of any clinical importance, and it is not yet known whether there are permanent alterations in pulmonary function after cardiac surgery. However, although modest, the postoperative changes in pulmonary function may be important for patients with pre-existing pulmonary diseases or those undergoing future thoracic surgery. Further studies are needed to identify possible risk factors for the development of long-term postoperative changes in pulmonary function after cardiac surgery. There is also a need for studies investigating the clinical relevance of the reduction of pulmonary function, as well as studies evaluating postoperative pulmonary management in the long-term perspective after cardiac surgery.

## Conclusion

Patients who undergo cardiac surgery have not fully recovered pulmonary function measured as static and dynamic lung volumes 1 year after cardiac surgery, while health-related quality of life are improved in comparison to preoperative values. A more pronounced decrease in pulmonary function measurements was associated with impairments regarding dyspnoea and subjective breathing and coughing ability. The reason for the postoperative pulmonary impairment is not known, and the clinical importance of a minor pulmonary impairment after cardiac surgery remains to be determined.

## Abbreviations

AoO, aortic occlusion; BMI, body mass index; BP, bodily pain; CABG, coronary artery bypass grafting; CI, confidence interval; ECC, extracorporeal circulation; FEV 1, forced expiratory volume in 1 s; FRC, functional residual capacity; FVC, forced vital capacity; GH, general health; HRQoL, health-related quality of life; IC, inspiratory capacity; LIMA, left internal mammary artery; Md, median; MH, mental health; mMRC, Modified Medical Research Council dyspnoea scale; NRS, numeric rating scale; NYHA, New York Heart Association classification; PEF, peak expiratory flow; PF, physical functioning; RE, emotional role functioning; RIMA, right internal mammary artery; RP, physical role functioning; RV, residual volume; SF, social functioning; SF-36, Short-Form Health Survey; SpO_2_, saturation of peripheral oxygen; TLC, total lung capacity; VC, vital capacity; VT, vitality
